# What do US and Canadian parents do to encourage or discourage physical activity among their 5-12 Year old children?

**DOI:** 10.1186/s12889-017-4918-z

**Published:** 2017-12-01

**Authors:** Andrew W. Tu, Teresia M. O’Connor, Mark R. Beauchamp, Sheryl O. Hughes, Tom Baranowski, Louise C. Mâsse

**Affiliations:** 1 Child & Family Research Institute, School of Population and Public Health, University of British Columbia, Vancouver, BC Canada; 2 USDA/ARS Children’s Nutrition Research Center, Baylor College of Medicine, Houston, TX USA; 3 School of Kinesiology, University of British Columbia, Vancouver, BC Canada

**Keywords:** Parenting, Parenting practices, Physical activity, Child, Qualitative

## Abstract

**Background:**

Parents have the potential to substantively influence their child’s physical activity. This study identified the parenting practices of US and Canadian parents to encourage or discourage their 5-12 year-old child’s physical activity and to examine differences in parenting practices by country, parental sex, age of child, and income.

**Methods:**

The sample consisted of 134 US and Canadian parents (54.5% US; 60.4% female) recruited from a web-based panel by a polling firm. The parents answered open-ended questions about what they and other parents do to encourage or discourage their child to be active. Responses were coded using a scheme previously developed to code items used in the published literature. Coded responses were summarized by domain and dimension with differences in responses by country, parental sex, age of child, or household income assessed with a log-linear analysis.

**Results:**

The 134 parents provided 649 and 397 responses to ways that parents encourage or discourage their child’s physical activity, respectively. Over 70% of responses for practices that encourage physical activity were related to structure of the environment, parental encouragement, and co-participation. The most common response was co-participation in activity with the child. Of the practices that discourage physical activity, 67% were related to structure of the environment, lack of parental control, and modeling poor behaviors. The most common response was allowing screen time. There were no differences in response by country, parental sex, child age, or household income.

**Conclusions:**

Parents most often encouraged physical activity through structure and emotional support and discouraged physical activity through lack of structure and control. Understanding how parents influence their child’s physical activity may help improve intervention strategies. The current results will inform the development of a physical activity parenting practices instrument.

**Electronic supplementary material:**

The online version of this article (10.1186/s12889-017-4918-z) contains supplementary material, which is available to authorized users.

## Background

High levels of physical activity during childhood have been linked to a number of health benefits including a reduction in blood pressure, blood lipid levels, body fat, and depressive symptoms and an improvement in bone density [1]. Despite these benefits, national physical activity levels are low. Recent estimates have found that less than 20% of Canadian and US children between the ages of 6 and 19 accumulate at least 60 min of physical activity per day [2, 3].

Parents have been identified as having the potential to substantively influence their child’s physical activity [4, 5]. For example, parents can provide emotional (e.g., praise, encouragement) or tangible (e.g., financial, transportation) support for physical activity; directly model physical activity; structure their child’s environment to promote physical activity; promote autonomous decision making regarding physical activity; or attempt to control their child’s behavior (e.g., through pressure or restriction) [6]. These parenting behaviors or practices were related to child physical activity levels, although the evidence was weak and mixed [7–9]. A systematic review of physical activity parenting practice studies revealed that parental support for physical activity was most consistently associated with child physical activity; however, support typically encompassed multiple domains (e.g., encouragement, co-participation, tangible support) [9]. In a longitudinal study, parental encouragement to be physically active was associated with children’s levels of physical activity 5 years later [10].

Few studies have qualitatively examined the parenting practices that parents commonly use to encourage or discourage their child to be physically active. Existing qualitative studies focused on specific ethnic populations [11–13]. Nominal group technique sessions were conducted with Chinese [11] and Hispanic [12] parents of preschoolers and found that parents most often reported parental engagement, logistic support, parental encouragement, and promoting other health behaviors as practices used to encourage physical activity and safety concerns, permissiveness of sedentary behavior, lack of time, psychological control, and emotional abuse as practices used to discourage physical activity. Among Australian Middle Eastern parents of 5 to 12 year old children, the majority of parents promoted physical activity through organized sports and encouraging outdoor play while focus on academic achievement and lack of time were reasons parents discouraged physical activity [13]. Therefore, there is need to gain a greater understanding in more diverse samples. Assessing the physical activity parenting practices among samples representative of the general US and Canadian populations can inform development of a parenting practice measure to fully understand how parenting affects health behaviors.

Building from our previous study which found that parents reported using physical activity parenting practices that are not included in current measures [6], this study qualitatively explored the parenting practices that may encourage or discourage physical activity in their children. This disconnect between parent reported practices and practices measured in the literature highlights the importance of collecting qualitative data to understand how parents influence their child’s behavior. The objective of this study was to identify the parenting practices that are predominantly reported by US and Canadian parents to encourage or discourage their child’s physical activity. In addition, this paper explored whether the parenting practices differed by: 1) parental sex and age of child as previous studies found differences in physical activity parenting practices for both of these indicators [9]; 2) income as this variable is a marker of resources which can be devoted to physical activity; and 3) country as it was thought important to examine the stability of the findings across the sample. This study opted to use income instead of educational background since educational background of both parents was not collected.

## Methods

This research protocol was approved by the Research Ethics Board of the University of British Columbia and received Institutional Review Board approval from the Baylor College of Medicine.

### Sample

The sample consisted of parents of 5-12 year old children who were living in Canada or the USA. Parents were recruited by an internet research polling firm (YouGovPolimetrix, US) from their web-based panel membership. Recruitment from a polling firm represented a cost-effective approach of obtaining a large representative sample over other sampling approaches. Recruitment occurred between November 2013 and February 2014. All panel members provided consent to be part of the panel and to participate in the survey. To be eligible, participants had to be the primary guardian of a 5 to 12 year old child. Participants were excluded if the child had a physical or learning disability that limited their child’s physically activity. Panel members were sampled to reflect the socio-economic and ethnic diversity of the two countries based on the 2012 US and 2011 Canadian census estimates. To reflect socio-economic diversity, country-specific household income cut-points (< 40th percentile, ≥40th to ≤80th percentile, and >80th percentile) were created and a corresponding percentage of participants (40%; 40%; 20%) were recruited into each group. To reflect ethnic diversity, participants were recruited based on the percentages of the largest ethnic groups for each country (White, Hispanic, Black, and other in the US and White, East/Southeast Asian, South/West Asian, and other in Canada). In addition, the sample was balanced between parents with younger (5-8 years of age) and older (9-12 years of age) children. In total, 134 parents (73 US and 61 Canadian) provided valid responses. Participants received 2000 points for their participation in this survey which could be redeemed as cash or gift cards valued at about $5 USD/Cdn.

### Questionnaire

Parents were asked to respond to a series of screening and socio-demographic questions to ensure eligibility. Those who met the eligibility criteria were asked to respond to the following four questions based on their youngest or oldest child within the age criteria (selected by the data collection program): 1) What sorts of things do you do to encourage your child to be physically active?; 2) What rules or guidelines do you have that may encourage your child to be physically active?; 3) What sorts of things might you do that may unintentionally affect your child from being physically active?; 4) Thinking about other parents with children of the same age, what things do they do that may discourage their children from being physically active? The fourth question asked parents to respond generally about other parents to avoid socially desirable responses. Each of the four questions was open-ended and parents could provide up to ten 160 character responses per question. A character limit was set to limit each response to one practice and to encourage parents to provide multiple responses (i.e., identify several parenting practices). Parents were prompted to expand on their responses if they provided short answers (<50 characters). As the questions were open-ended, parents were blocked from completing the questionnaire on their mobile devices which may limit their ability to type more detailed responses. The online survey was piloted among 25 Canadian parents using cognitive interviewing techniques [14]. Responses from questions 1 and 2 were grouped and categorized as encouraging parenting practices and responses from questions 3 and 4 were grouped and categorized as discouraging parenting practices.

### Coding of responses

Detailed information about the development of the coding scheme used in this study can be found elsewhere [6]. Briefly, the coding scheme was initially developed to code 74 published questionnaires/instruments designed to assess physical activity parenting practices, which included 608 items [6]. The coding scheme was developed based on a review of these published constructs, associated items and was informed by conceptual frameworks of parenting practices for physical activity and nutrition [15, 16]. The coding scheme consisted of 6 broad domains, 14 dimensions, and 1 to 5 sub-dimensions [6]. The domains (dimensions in parenthesis) were structuring of the activity environment (monitoring, structure of the environment); emotional support (expressing positive emotions, parental encouragement); parental control (expressing negative emotions, lack of parental control, pressure to be active, restriction, rewards and discipline); informational support (modeling, teach/reason); autonomy promotion (autonomy support, co-participation); and tangible support (logistic support/facilitation) (see our previous study for the full coding scheme and detailed definitions) [6]. Each item from the 74 instruments was assigned to a dimension and sub-dimension in order to group similar items. The list of items was further reduced using a winnowing process that grouped similar items into a statement that best captured the parenting practice (608 items winnowed to 100 parenting practice statements). For example, three published items asking about parent co-participation in physical activity (“During a typical week, how often has a female adult done physical activity with you?”; “How often do you exercise with one or both of your parents?”; and “How often do you do the following activities together as a family with at least one adult family member – Play sports?”) were reduced to the following generic statement “Participate in [physical activity, sports, exercise] or play active games with my child.”

Each parent response to the four questions on the online questionnaire was coded to a parenting practice from the consolidated list of 100 parenting practice statements. For example, when parents were asked what they did to encourage their child to be physically active, one parent responded “Ask if she wants to join sports at school, usually she does not” which was coded to the item “I allow my child to choose whether s/he participates in sports or vigorous physical activity in his/her free time.” As the item was meant to describe a concept, responses in a negative direction could have been coded to an item in the opposite direction, if that already existed in the coding scheme. For example, one parent’s response to a question asking what the other parents did to discourage their child from being physically active responded “Not participating with their kids at all” which was coded to the item “I participate in [activity type] with my child.” If the parent response had elements of two distinct items, then the response was coded to both items. Coding was conducted independently by two researchers, discrepancies were discussed, and a consensus was reached. A third researcher reviewed the coding and discussed discrepancies with the other two researchers. A new code was created for any unique parenting item that did not appear in the literature. If more than one response from the same parent was coded to the same item, the additional responses were removed to avoid repetition.

### Analysis

Coded responses to the encouraging and discouraging questions were ranked by domain and dimension. To assess whether there were differences in responses by country, parental sex, age of child, or household income, a log-linear analysis with iterative proportional fitting was conducted. Log-linear analysis is an iterative process which can test for higher order (e.g., three-way) associations among categorical variables. Analyses were conducted on the above four sets of variables with each set including the coded domain (6 categories), a dichotomous variable indicating whether the response was encouraging or discouraging (2 categories), and one of country (USA, Canada), parental sex (male, female), age of child (5-8, 9-12), or household income (below median, above median). All two- and three-way interactions were assessed within each set. For each set, a saturated model was formed and each higher order term was sequentially removed to examine the goodness-of-fit. Model fit was based on Pearson’s Chi-square statistic (χ^2^) and the deviance statistic (G^2^). Models with significant goodness-of-fit statistics suggest poor fit. The most parsimonious model (model with least number of interaction terms) was chosen as the best fitting model for each set. A *p*-value of <0.05 was considered significant. Stata (version 13.1, College Station, Texas) was used for all analyses.

## Results

Participant characteristics can be found in Table 1. Of the 134 participants, 73 (54.5%) were from the US and 81 (60.4%) were female. On average, each parent provided 4.8 (SD 2.5; range 0-13) responses to practices that encourage physical activity and 3.0 (SD 1.9; range 0-11) responses to practices that discourage physical activity. In total, from the 134 parents, 649 responses were practices that parents said encouraged their child to engage in physical activity (coded to 78 unique parenting practices) and 397 responses were practices that discouraged physical activity (coded to 69 unique parenting practices). In addition, 27 of the parenting practices (17 encouraging and 10 discouraging practices) did not link to any of the 100 parenting practices statements found in the published measures of physical activity parenting (see Additional file 1: Appendices 1 and 2 for parenting practices not included in the literature).

**Table 1:** Participant characteristics

	USA (*n* = 73)	Canada (*n* = 61)
Parent Sex (female)	43 (58.9)	38 (62.3)
Age of child		
5-8	33 (45.2)	32 (52.5)
9-12	40 (54.8)	29 (47.5)
Marital status		
Married or common-law	54 (74.0)	50 (82.0)
Separated or divorced	11 (15.1)	8 (13.1)
Never married	7 (9.6)	3 (4.9)
Widowed	1 (1.4)	0
Education		
High school or less	22 (30.1)	5 (8.2)
Certificate/diploma/or some college or university education	13 (17.8)	16 (26.2)
Bachelor’s degree	22 (30.1)	29 (47.5)
Postgraduate degree	16 (21.9)	11 (18.0)
Income		
Below median	37 (50.7)	35 (57.4)
Above median	32 (43.8)	26 (42.6)
Missing	4 (5.5)	0

### Parenting practices that encourage physical activity

About two-thirds of the responses to ways parents encouraged physical activity were coded to structure of the activity environment and emotional support (see Table 2). The ten most coded responses are displayed in Table 3 and represent 50% of all responses related to encouraging physical activity. The full list of coded responses can be found in Additional file 1: Appendix 1. The percentage of participants that mentioned each of the top 10 parenting practices ranged from 15.7% to 33.6%. The top 10 practices used to encourage physical activity included co-participation (participating in physical activity or walks with child), parental encouragement (encouraging outdoor play, physical activity, biking or walking in the neighborhood), structure of the environment (restricting sedentary behavior, ensuring active transport, taking child to park or play spaces), and tangible support (enrolling child in physical activity).

**Table 2:** Responses by domain and dimension

	Encourage (n = 649)	Discourage (n = 397)
Structure of the activity environment	236 (36.4)	154 (38.8)
Monitoring	15 (2.3)	1 (0.3)
Structure of the environment	221 (34.1)	153 (38.5)
Emotional support	183 (28.2)	15 (3.8)
Expressing positive emotions	8 (1.2)	5 (1.3)
Parental Encouragement	175 (27.0)	10 (2.5)
Parental control	32 (4.9)	113 (28.5)
Lack of parental control	2 (0.3)	65 (16.4)
Expressing negative emotions	6 (0.9)	8 (2.0)
Pressure to be active	11 (1.7)	2 (0.5)
Restriction	1 (0.2)	34 (8.6)
Rewards and discipline	12 (1.9)	4 (1.0)
Informational support	50 (7.7)	50 (12.6)
Modeling	21 (3.2)	49 (12.3)
Teach/reason	29 (4.5)	1 (0.3)
Autonomy promotion	84 (12.9)	19 (4.8)
Autonomy support	8 (1.2)	8 (2.0)
Co-participation	76 (11.7)	11 (2.8)
Tangible support	64 (9.9)	46 (11.6)

**Table 3:** Top 10 coded responses to parenting practices that encourage physical activity (n = 649)

Coded Response	Domain	Dimension	Number of responses	Percent of responses	Percent of participants	Sample parent responses
I participate in [activity type] or play active games with my child.	Autonomy Promotion	Co-participation	45	6.9	33.6	“We ride bike every afternoon” “When it is warm, we play tag outside” “We will wrestle around the house”
When the weather is nice, I encourage my child to play outside.	Emotional Support	Parental Encouragement	40	6.2	29.9	“Weather permitting, put them outside in the yard for set periods of time” “We encourage her to go outside” “I encourage her to invite her friends over to play outside or go to the park”
I limit the amount of time my child spends [sedentary activity type] on weekend/weekday.	Structure of the Activity Environment	Structure of the Environment	39	6.0	29.1	“We limit her computer and TV time” “Cut back video games to only weekends” “They only have 2 h a day of video games, the rest either outside or reading”
I enroll my child in [activity type].	Tangible Support	Logistic Support/ Facilitation	37	5.7	27.6	“She is signed up in swimming and tennis classes” “Sign him up to soccer, hockey, and basketball clubs to ensure that they get enough activity” “Enroll him in a fitness program”
I encourage my child to participate in physical activity, or play sports (/in his/her free time).	Emotional Support	Parental Encouragement	34	5.2	25.4	“Encourage him to participate in school sports” “Play games with other siblings” “Encourage him to play at school playgrounds instead of staying inside the classes after lunch”
I encourage my child to ride a bike and walk in our neighborhood to be active.	Emotional Support	Parental Encouragement	33	5.1	24.6	“They love riding bikes so I encourage that” “Go for walks, walk the dog” “Allow her to go outside to ride her bike and scooter”
I make sure my child uses active transportation to go to school (e.g., walk, bicycle, use public transportation).	Structure of the Activity Environment	Structure of the Environment	27	4.2	20.1	“She walks to and from school with her brother” “My kids walk to and from the bus stop every school day” “Walk to school when we can”
I take my child to the park, playground, or places that s/he can be physically active.	Structure of the Activity Environment	Structure of the Environment	26	4.0	19.4	“I take her to the park to play” “I take him to the skating rink” “Go to the pool 3-4 times a week”
I make sure my child uses active transportation to do errands close to home or to go places close to home such as by walking or bicycling.	Structure of the Activity Environment	Structure of the Environment	24	3.7	17.9	“We do light shopping on foot and heavy shopping by car” “I encourage him to use a bicycle to go to close places” “We walk to the library when the weather allows”
I go for walks with my child.	Autonomy Promotion	Co-participation	21	3.2	15.7	“She goes for walks with me” “Usually, we love to walk after meals” “Take them for walks with me every evening”

### Parenting practices that discourage physical activity

About two-thirds of responses to ways parents discourage physical activity were coded to the structure of the activity environment and parental control domains with structure, lack of parental control, and restriction being the dominant dimensions (Table 2). The top 10 most coded responses are displayed in Table 4 and make up 60% of all responses to parenting practices that discourage physical activity (see Additional file 1: Appendix 2 for entire list). The most common practice reported was lack of parental control (allow child to watch TV or play video/computer games whenever s/he wants) which represented 15.9% of all responses to ways parents discourage physical activity and was mentioned by 47% of participants. The remaining parenting practices within the top 10 were mentioned by 7.5% to 20.1% of the participants. Coded responses in the top 10 included practices related to structure of the environment (requiring supervision when outdoors, restricting outdoor play, restricting physical activity indoors), restriction (due to potential injury), modeling (child sees me being sedentary), and tangible support (lack of time).

**Table 4:** Top 10 coded responses to parenting practices that discourage physical activity (n = 397)

Coded Response	Domain	Dimension	Number of responses	Percent of responses	Percent of participants	Sample parent responses
I allow my child to watch TV or play video/computer games whenever s/he wants to.	Parental Control	Lack of parental control	63	15.9	47.0	“I let her watch more TV or use the computer more than she should due to other demands around the house” “Letting their children rely on too many electronic devices” “Allow too much time on the computer/TV”
My child must be supervised when s/he is active outside.	Structure of the Activity Environment	Structure of the Environment	27	6.8	20.1	“I don’t let her go outside alone” “If I can’t monitor you, you can’t participate in that” “She does not ride her bike outside alone except when an adult is with her”
I don’t allow my child to play outside in the street after dark or after a certain time.	Structure of the Activity Environment	Structure of the Environment	26	6.5	19.4	“He is to come inside before it gets too dark outside” “Not allowing children out after dark” “She can only play inside after dark”
My child sees me being sedentary (e.g. watching TV, on the computer, sleeping a lot).	Informational Support	Modeling	26	6.5	19.4	“We’re both out of shape and watch a lot of TV at night laying on the couch” “I am on the computer often” “Stay inside and do nothing ourselves”
I [am/have enough time to be] involved in my child’s activities (e.g. coaching activities, watching child play).	Tangible Support	Logistic Support/ Facilitation	22	5.5	16.4	“Too tired/busy to play with them outside” “Not taking enough time or effort to care for their children” “Our lifestyle for now prevent him to be more active”
I restrict some physical activities because I am afraid my child will be hurt.	Parental Control	Restriction	20	5.0	14.9	“I don’t let my child ride her scooter as much as she would like because I am scared she will get hurt” “Bubble-wrapping them by not letting them explore their environments” “Discouraging him to run outside during winter for fear of slipping and falling”
I don’t allow my child to play outside in bad weather. (*)	Structure of the Activity Environment	Structure of the Environment	13	3.3	9.7	“If it is too cold, he is not allowed outside” “We don’t allow our children to play in the rain” “I do not allow my child to play outside when it is too cold/hot”
I restrict the amount of time my child spends playing outside.	Structure of the Activity Environment	Structure of the Environment	12	3.0	9.0	“Not allow them to play outside with other kids” “He’s always sick and I don’t let him outside” “Keep them indoors versus taking them outdoors”
I have rules that my child is not allowed to walk [e.g., to the neighborhood park] alone.	Structure of the Activity Environment	Structure of the Environment	10	2.5	7.5	“I won’t let her walk alone” “Do not allow him to go to the park on his own” “Don’t let their kids walk anywhere on their own”
I restrict [activity type] inside the house.	Structure of the Activity Environment	Structure of the Environment	10	2.5	7.5	“I won’t allow them to run in the house” “Don’t let them install chin-up bars in the doorway” “Stop them from playing when I am tired”

Log linear analyses were conducted to examine whether the responses differed by country, parental sex, age of child, and household income. Results of the log-linear analyses found that the best fitting model in all four cases was one that only included an interaction between dimension and encouraging/discouraging parenting practices (Table 5). Specifically, the interaction found that parents emphasized different dimensions depending on whether they wanted to encourage or discourage their child to be physically active. The lack of an interaction with any of the socio-demographic variables indicates no differences were found in the responses by country, parental sex, age of child, or income.

**Table 5:** Results of the log linear analysis to test for differences in responses by parental sex, country, age of child, and household income

	G^2^	X^2^	df	p-value
Model 1: Parental sex				
(D, P, S)	235.9	213.1	16	<0.001
(D, PS)	235.7	212.8	15	<0.001
(P, DS)	226.4	205.4	11	<0.001
(S, DP)	16.4	17.0	11	0.126
(DP, DS)	6.86	6.92	6	0.334
(DP, PS)	16.2	16.7	10	0.093
(DS, PS)	226.2	205.0	10	<0.001
(DP, DS, PS)	6.59	6.70	5	0.253
(DPS)	0	0	0	
Model 2: Country				
(D, P, C)	228.7	209.4	16	<0.001
(D, PC)	228.7	209.4	15	<0.001
(P, DC)	225.3	205.2	11	<0.001
(C, DP)	9.22	9.22	11	0.601
(DP, DC)	5.80	5.82	6	0.446
(DP, PC)	9.17	9.17	10	0.516
(DC, PC)	225.3	205.2	10	<0.001
(DP, DC, PC)	5.79	5.81	5	0.327
(DPC)	0	0	0	
Model 3: Age of child				
(D, P, A)	228.7	208.3	16	<0.001
(D, PA)	228.7	208.3	15	<0.001
(P, DA)	222.5	201.5	11	<0.001
(A, DP)	9.20	9.12	11	0.604
(DP, DA)	2.95	2.93	6	0.815
(DP, PA)	9.19	9.12	10	0.514
(DA, PA)	222.5	201.5	10	<0.001
(DP, DA, PA)	2.81	2.78	5	0.729
(DPA)	0	0	0	
Model 4: Household income				
(D, P, I)	233.7	209.7	16	<0.001
(D, PI)	233.6	209.4	15	<0.001
(P, DI)	224.9	203.3	11	<0.001
(I, DP)	17.5	17.5	11	0.093
(DP, DI)	8.75	8.70	6	0.188
(DP, PI)	17.5	17.5	10	0.065
(DI, PI)	224.8	203.2	10	<0.001
(DP, DI, PI)	8.74	8.69	5	0.120
(DPI)	0	0	0	

*D* Dimension (6 categories), *P* Parenting behavior (Encourage/Discourage), *S* Parental sex (M/F), *C* Country (USA/Canada), *A* Age of child (5-8/9-12), *I* Household income (below median/above median). XY represents two-way interactions; XYZ represents three-way interactions

## Discussion

This study examined the parenting practices US and Canadian parents used to encourage and discourage physical activity. While the majority of the practices reported by the parents were captured in published research instruments, this study uncovered 27 unique parenting practices that were not captured (see Additional file 1: Appendix). This highlights the importance of conducting qualitative research in gaining a better understanding of parent’s beliefs and health behaviors.

The most emphasized parenting practices used to encourage physical activity among children include co-participation in physical activity, encouraging physical activity or outdoor play, limiting sedentary behavior, enrolling children in physical activity classes or lessons, ensuring children use active transportation to go places, and taking children to the park or play spaces. Similar to other studies, co-participation in physical activity was the most endorsed parenting practice used to encourage physical activity [11, 12]. Previous studies have found inconsistent associations between parent-child co-participation in physical activity and child physical activity [17–19]; however, co-participation has also been included in a higher domain of parental support or encouragement which has been frequently associated with child physical activity [9]. The remaining parenting practices used to encourage physical activity were dominated by two domains: structure of the environment and parental encouragement. Structure refers to ways parents set up the environment in the home to influence their child’s physical activity whereas parental encouragement refers to the various ways parents encourage children to participate in physical activity [6]. The frequency of reporting of these parenting practices suggests that parents may be more accepting to use these practices to encourage physical activity. Future interventions should explore whether promoting these practices will influence child physical activity behavior.

The most common parenting practices used by Canadian and US parents to discourage physical activity included allowing sedentary behavior, limiting outdoor time due to lack of supervision, darkness, or poor weather, modeling poor behaviors, lack of time, restricting physical activity due to injury, restricting the time spent outdoors, and restricting indoor physical activity. Similar to past studies, allowing sedentary behavior was the most endorsed practice to discourage physical activity [11, 12].. This practice was reported by almost half of the participants which was more than double the number of participants who reported the second-most endorsed practice used to discourage physical activity. Parental permissiveness of sedentary behaviors has been linked to increase screen time [20] but its association with physical activity is unclear. Permissive parenting in general has been associated with higher amounts of physical activity among children [21, 22]. Practices related to structure of the environment were also heavily emphasized by parents as practices used to discourage physical activity. The most common practices related to structure involved restriction or rules surrounding outdoor time. There is evidence that outdoor play is declining in North America [23, 24]. Active outdoor play is important for healthy development of children and parents should encourage their children to explore their environment [25].

There were no significant differences in the reporting of parenting practices used to encourage or discourage physical activity by domain and each of the four socio-demographic variables tested (country, parental sex, age of child, and income).The results are in contrast with studies that have found differences in parenting practices by parental sex [26] and income [11]; however, these studies were conducted in countries other than Canada and the US. Lack of differences in our results among these four socio-demographic variables suggests that the findings of this study may be generalizable to the larger Canada and US population.

This study is not without limitations. The web-based platform used to collect parent responses only allowed for structured questions. Therefore, there was no opportunity to ask parents to elaborate on specific responses or to probe further the meaning of their responses. Further discussions with parents may have uncovered more parenting practices used to encourage or discourage physical activity. In addition, the participants for this study were sampled from a web-based panel and may not be representative of US and Canadian parents. However, a quota sampling approach was used to ensure an income and ethnic distribution that matched the income and ethnic distribution of the US and Canadian population. Furthermore, information about the child’s sex was not collected; therefore, we could not examine differences in parenting practices by child sex. Finally, child physical activity behavior was not measured; therefore, the extent to which specific parenting practices influence child behavior cannot be ascertained from this study.

## Conclusions

This study explored common parenting practices used by US and Canadian parents to encourage or discourage physical activity among their children. Parents most often encouraged physical activity through structure and emotional support and discouraged physical activity through lack of structure and control. The findings provide a unique understanding of the approaches used by parents to influence their child’s physical activity and highlight the importance of using qualitative methods to uncover parental beliefs and behaviors. Understanding the different behaviors used by parents to influence physical activity will help in the development of an instrument to measure parenting practices related to physical activity. Future studies are needed to ascertain whether these practices used by parents affect child physical activity behavior.

## Additional file

Additional file 1: Appendix 1. Coded responses to parenting practices that encourage physical activity. **Appendix 2.** Coded responses to parenting practices that discourage physical activity. (DOCX 26 kb)
