# Supplementary material for: What do US and Canadian parents do to encourage or discourage physical activity among their 5-12 Year old children?
Source: BMC Public Health. 2017 Dec 1;17:920. doi: 10.1186/s12889-017-4918-z (PMC5710093; doi:10.1186/s12889-017-4918-z)
Supplement: Additional file 1: Appendix 1. — Coded responses to parenting practices that encourage physical activity. Appendix 2. Coded responses to parenting practices that discourage physical activity. (DOCX 26 kb) [file 12889_2017_4918_MOESM1_ESM.docx]

Appendix 1: Frequency of coded responses to parenting practices that encourage physical activity (n=649)

| **Coded Responses** | **Domain** | **Dimension** | **N (%)** |
| --- | --- | --- | --- |
| I participate in [activity type] or play active games with my child. | Autonomy Promotion | Modeling – co-participation | 45 (6.9) |
| When the weather is nice, I encourage my child to play outside. | Emotional Support | Parental Encouragement | 40 (6.2) |
| I limit the amount of time my child spends [sedentary activity type] on weekend/weekday. | Structure of the Activity Environment | Structure of the Activity Environment | 39 (6.0) |
| I enroll my child in [activity type]. | Tangible Support | Logistic support/facilitation | 37 (5.7) |
| I encourage my child to participate in physical activity, or play sports (/in his/her free time). | Emotional Support | Parental Encouragement | 34 (5.2) |
| I encourage my child to ride a bike and walk in our neighborhood to be active. | Emotional Support | Parental Encouragement | 33 (5.1) |
| I make sure my child uses active transportation to go to school (e.g., walk, bicycle, use public transportation). | Structure of the Activity Environment | Structure of the Activity Environment | 27 (4.2) |
| I take my child to the park, playground, or places that s/he can be physically active. | Structure of the Activity Environment | Structure of the Activity Environment | 26 (4.0) |
| I make sure my child uses active transportation to do errands close to home or to go places close to home such as by walking or bicycling. | Structure of the Activity Environment | Structure of the Activity Environment | 24 (3.7) |
| I go for walks with my child. | Autonomy Promotion | Modeling – co-participation | 21 (3.2) |
| I use sport/physical activity as a form of family recreation (e.g., going on bike rides together, hiking, skating). | Structure of the Activity Environment | Structure of the Activity Environment | 17 (2.6) |
| If the weather is nice, my child knows that s/he is expected to play outside. | Structure of the Activity Environment | Structure of the Activity Environment | 16 (2.5) |
| I have a rule that my child must participate in active sports or physical activities. | Structure of the Activity Environment | Structure of the Activity Environment | 15 (2.3) |
| I provide equipment (like soccer balls, basketballs, sporting goods, or active video games) for my child to be physically active. | Tangible Support | Logistic support/facilitation | 15 (2.3) |
| I use my own behavior to encourage my child to be physically active. | Informational Support | Modeling – healthy/unhealthy | 14 (2.2) |
| I encourage my child to use resources in our neighborhood to be active (such as the park, community centres, or school facilities). | Emotional Support | Parental Encouragement | 13 (2.0) |
| I involve my child in active chores and yard work around the house. | Structure of the Activity Environment | Structure of the Activity Environment | 13 (2.0) |
| I tell my child that physical activity or vigorous exercise is good for his/her health. | Informational Support | Teach/Reason | 13 (2.0) |
| I make sure my child is physically active at least 60 minutes per day. | Structure of the Activity Environment | Monitoring | 11 (1.7) |
| I invite my child to join me if I exercise or do something active. | Autonomy Promotion | Modeling – co-participation | 10 (1.5) |
| I tell my child to exercise or play sports. | Parental Control | Pressure to be Active | 10 (1.5) |
| I try to make physical activity into a fun game to get my child to be more active. (*) | Emotional Support | Parental Encouragement | 10 (1.5) |
| My child is responsible for taking the dog for a walk and/or playing with the dog. (*) | Structure of the Activity Environment | Structure of the Activity Environment | 10 (1.5) |
| I encourage my child to be active for at least 60 min per day. | Emotional Support | Parental Encouragement | 9 (1.4) |
| I reward my child for exercising. | Parental Control | Rewards and Discipline | 9 (1.4) |
| I arrange for my child to be with friends in order to be active with them. (*) | Structure of the Activity Environment | Structure of the Activity Environment | 7 (1.1) |
| I encourage active video games as a way to be active indoors. (*) | Emotional Support | Parental Encouragement | 7 (1.1) |
| I encourage competition during activities to get my child to be more active. (*) | Emotional Support | Parental Encouragement | 7 (1.1) |
| My child sees me being physically active (walking, cycling, playing sports). | Informational Support | Modeling – healthy/unhealthy | 7 (1.1) |
| I expect my child to stick with his/her physical activity or sports. | Structure of the Activity Environment | Structure of the Activity Environment | 6 (0.9) |
| I practice active habits with my child (e.g. parking far from the door, taking the stairs). (*) | Structure of the Activity Environment | Structure of the Activity Environment | 6 (0.9) |
| I pressure my child to try harder at sports or his/her physical activity. | Parental Control | Expressing Negative Emotions | 6 (0.9) |
| I spend time teaching my child how to play a sport or do certain physical activities. | Informational Support | Teach/Reason | 5 (0.8) |
| I take my child to dance lessons, physical activity training lessons, or sporting practices. | Tangible Support | Logistic support/facilitation | 5 (0.8) |
| I try to get my child to be active (e.g. playing tag, biking, dancing) instead of watching TV or playing video games. | Emotional Support | Parental Encouragement | 5 (0.8) |
| I encourage my child to walk instead of riding in a stroller or being carried. | Structure of the Activity Environment | Structure of the Activity Environment | 4 (0.6) |
| I provide my child with physical activity options from which my child can choose. | Autonomy Promotion | Autonomy Support | 4 (0.6) |
| I restrict [activity type] inside the house. | Structure of the Activity Environment | Structure of the Activity Environment | 4 (0.6) |
| I talk with my child about his/her (exercise, physical activity, sports). | Emotional Support | Parental Encouragement | 4 (0.6) |
| I get my child to be physically active by telling him/her s/he will have fun doing the activity or sport. (*) | Emotional Support | Expressing Positive Emotions | 3 (0.5) |
| I praise my child for being physically active or for participating in sports. | Emotional Support | Expressing Positive Emotions | 3 (0.5) |
| I set physical activity challenges for my child (e.g. walking a certain distance). (*) | Emotional Support | Parental Encouragement | 3 (0.5) |
| I support my child in being physically active. | Emotional Support | Parental Encouragement | 3 (0.5) |
| I watch sports with my child or take my child to sports games to encourage him/her to participate in physical activity. (*) | Informational Support | Teach/Reason | 3 (0.5) |
| I allow my child to watch TV or play video/computer games whenever s/he wants to. | Parental Control | Child Control | 2 (0.3) |
| I allow my child to choose whether s/he participates in sports or vigorous physical activity in his/her free time. | Autonomy Promotion | Autonomy Support | 2 (0.3) |
| I encourage my child to be less sedentary. | Emotional Support | Parental Encouragement | 2 (0.3) |
| I have a rule 'If you want a treat, you need to exercise.' (*) | Structure of the Activity Environment | Structure of the Activity Environment | 2 (0.3) |
| I have rules that my child needs to wear protective equipment (e.g., bike helmet, knee pads) when being active. | Structure of the Activity Environment | Structure of the Activity Environment | 2 (0.3) |
| I have to make sure my child gets enough exercise. | Structure of the Activity Environment | Monitoring | 2 (0.3) |
| I keep track of the amount of physical activity or exercise my child gets. | Structure of the Activity Environment | Monitoring | 2 (0.3) |
| I reward my child for good behavior with TV, DVD, or computer time. | Parental Control | Rewards and Discipline | 2 (0.3) |
| I tell my child that [sedentary activity type] is not good for his/her (health/eyesight). | Informational Support | Teach/Reason | 2 (0.3) |
| I tell my child that physical activity will make him/her feel good and have more energy. (*) | Informational Support | Teach/Reason | 2 (0.3) |
| I try to encourage my child to do physical activities by telling my child that he/she has the potential to be an athlete. (*) | Emotional Support | Parental Encouragement | 2 (0.3) |
| In my home, we have (cable/satellite TV, computer, video games). | Tangible Support | Logistic support/facilitation | 2 (0.3) |
| I [am/have enough time to be] involved in my child's activities (e.g. coaching activities, watching child play). | Tangible Support | Logistic support/facilitation | 1 (0.2) |
| I do not enroll my child in physical activities that are too expensive. | Tangible Support | Logistic support/facilitation | 1 (0.2) |
| I don't allow my child to play on community or sports teams (/so s/he can concentrate on schoolwork). | Parental Control | Restriction | 1 (0.2) |
| I encourage my child to play board games to reduce his/her time spent playing video games or watching TV. | Emotional Support | Parental Encouragement | 1 (0.2) |
| I enroll my child in sport, dance or other physical activity program that s/he asks to be signed up for. | Tangible Support | Logistic support/facilitation | 1 (0.2) |
| I go to my child's activities and watch my child participate in [activity type].] | Emotional Support | Parental Encouragement | 1 (0.2) |
| I have a rule about not playing in the street. | Structure of the Activity Environment | Structure of the Activity Environment | 1 (0.2) |
| I have to nag my child to be physically active or more active. (*) | Parental Control | Pressure to be Active | 1 (0.2) |
| I help my child in every way I can when it comes to sports and exercises. | Tangible Support | Logistic support/facilitation | 1 (0.2) |
| I negotiate with my child on how much physical activity/sports s/he does. | Autonomy Promotion | Autonomy Support | 1 (0.2) |
| I negotiate with my child on how much TV/video/DVD s/he is allowed to watch. | Autonomy Promotion | Autonomy Support | 1 (0.2) |
| I punish my child by not allowing him/her to take part in physical activity or sports. | Parental Control | Rewards and Discipline | 1 (0.2) |
| I restrict the amount of time my child spends playing outside. | Structure of the Activity Environment | Structure of the Activity Environment | 1 (0.2) |
| I show my child examples of role models (i.e. people who are active) to encourage him/her to be active. (*) | Informational Support | Teach/Reason | 1 (0.2) |
| I store my child's active toys/sports equipment in a place that is easily accessible for my child. | Tangible Support | Logistic support/facilitation | 1 (0.2) |
| I tell my child how much I like to exercise, be physically active and/or play sports. | Emotional Support | Expressing Positive Emotions | 1 (0.2) |
| I tell my child s/he is doing well in physical activities or sports. | Emotional Support | Expressing Positive Emotions | 1 (0.2) |
| I tell my child that physical activity will make him/her look good. (*) | Informational Support | Teach/Reason | 1 (0.2) |
| I tell my child that watching TV/video/DVD can make him/her fat. | Informational Support | Teach/Reason | 1 (0.2) |
| I tell my child the long-term benefits of learning a new sport and staying active as it helps him/her later in life. (*) | Informational Support | Teach/Reason | 1 (0.2) |
| I try to encourage my child to do physical activities by telling them they will make new friends. (*) | Emotional Support | Parental Encouragement | 1 (0.2) |
| My child has rules to follow for physical activity such as being home at a set time, not going to some places, etc. | Structure of the Activity Environment | Structure of the Activity Environment | 1 (0.2) |

* New parenting items not found in the literature

Appendix 2: Frequency of coded responses to parenting practices that discourage physical activity (n=397)

| **Coded Responses** | **Domain** | **Dimension** | **N (%)** |
| --- | --- | --- | --- |
| I allow my child to watch TV or play video/computer games whenever s/he wants to. | Parental Control | Child Control | 63 (15.9) |
| My child must be supervised when s/he is active outside. | Structure of the Activity Environment | Structure of the Activity Environment | 27 (6.8) |
| I don't allow my child to play outside in the street after dark or after a certain time. | Structure of the Activity Environment | Structure of the Activity Environment | 26 (6.5) |
| My child sees me being sedentary (e.g. watching TV, on the computer, sleeping a lot). | Informational Support | Modeling | 26 (6.5) |
| I [am/have enough time to be] involved in my child's activities (e.g. coaching activities, watching child play). | Tangible Support | Logistic support/facilitation | 22 (5.5) |
| I restrict some physical activities because I am afraid my child will be hurt. | Parental Control | Restriction | 20 (5.0) |
| I don't allow my child to play outside in bad weather. (*) | Structure of the Activity Environment | Structure of the Activity Environment | 13 (3.3) |
| I restrict the amount of time my child spends playing outside. | Structure of the Activity Environment | Structure of the Activity Environment | 12 (3.0) |
| I have rules that my child is not allowed to walk to [e.g., the neighborhood park] alone. | Structure of the Activity Environment | Structure of the Activity Environment | 10 (2.5) |
| I restrict [activity type] inside the house. | Structure of the Activity Environment | Structure of the Activity Environment | 10 (2.5) |
| I use my own behavior to encourage my child to be physically active. | Informational Support | Modeling | 10 (2.5) |
| I participate in [activity type] or play active games with my child. | Autonomy Promotion | Modeling | 9 (2.3) |
| I restrict my child's outdoor activities because my neighborhood is not safe. | Parental Control | Restriction | 8 (2.0) |
| My child sees me being physically active (walking, cycling, playing sports). | Informational Support | Modeling | 8 (2.0) |
| I allow my child to choose whether s/he participates in sports or vigorous physical activity in his/her free time. | Autonomy Promotion | Autonomy Support | 7 (1.8) |
| I enroll my child in [activity type]. | Tangible Support | Logistic support/facilitation | 7 (1.8) |
| I make sure my child uses active transportation to do errands close to home or to go places close to home such as by walking or bicycling. | Structure of the Activity Environment | Structure of the Activity Environment | 6 (1.5) |
| My child has rules to follow for physical activity such as being home at a set time, not going to some places, etc. | Structure of the Activity Environment | Structure of the Activity Environment | 6 (1.5) |
| I do not enroll my child in physical activities that are too expensive. | Tangible Support | Logistic support/facilitation | 5 (1.3) |
| I don't allow my child to play on community or sports teams (./so s/he can concentrate on schoolwork). | Parental Control | Restriction | 5 (1.3) |
| I have a rule about not playing in the street. | Structure of the Activity Environment | Structure of the Activity Environment | 5 (1.3) |
| I make sure my child uses active transportation to go to school. (e.g., walk, bicycle, use public transportation). | Structure of the Activity Environment | Structure of the Activity Environment | 5 (1.3) |
| In my home, we have (cable/satellite TV, computer, video games). | Tangible Support | Logistic support/facilitation | 5 (1.3) |
| When my child plays outside, s/he must stay close by. | Structure of the Activity Environment | Structure of the Activity Environment | 5 (1.3) |
| I [sedentary activity] with child. (e.g., watch TV; play video games) | Informational Support | Modeling | 4 (1.0) |
| I limit the amount of time my child spends [sedentary activity] on weekend/weekday. | Structure of the Activity Environment | Structure of the Activity Environment | 4 (1.0) |
| I punish my child by not allowing him/her to take part in physical activity or sports. | Parental Control | Rewards and Discipline | 4 (1.0) |
| I take my child to the park, playground, or places that s/he can be physically active. | Structure of the Activity Environment | Structure of the Activity Environment | 4 (1.0) |
| I criticize or tell my child s/he is not good when doing certain sports. | Parental Control | Expressing Negative Emotions | 3 (0.8) |
| I encourage my child to ride a bike and walk in our neighborhood to be active. | Emotional Support | Parental Encouragement | 3 (0.8) |
| I pressure my child to try harder at sports or his/her physical activity. | Parental Control | Expressing Negative Emotions | 3 (0.8) |
| I take my child to dance lessons, physical activity training lessons, or sporting practices. | Tangible Support | Logistic support/facilitation | 3 (0.8) |
| I arrange for my child to be with friends in order to be active with them. (*) | Structure of the Activity Environment | Structure of the Activity Environment | 2 (0.5) |
| I encourage my child to participate in physical activity, or play sports (./in his/her free time). | Emotional Support | Parental Encouragement | 2 (0.5) |
| I enroll my child in sport, dance or other physical activity program that s/he asks to be signed up for. | Tangible Support | Logistic support/facilitation | 2 (0.5) |
| I enroll my child in too many activities leaving no time for free play. (*) | Tangible Support | Logistic support/facilitation | 2 (0.5) |
| I expect my child to stick with his/her physical activity or sports. | Structure of the Activity Environment | Structure of the Activity Environment | 2 (0.5) |
| I have a rule that my child must do homework before s/he is able to exercise or be physically active. | Structure of the Activity Environment | Structure of the Activity Environment | 2 (0.5) |
| I have a rule that my child must participate in active sports or physical activities. | Structure of the Activity Environment | Structure of the Activity Environment | 2 (0.5) |
| I have rules that my child needs to wear protective equipment (e.g., bike helmet, knee pads) when being active. | Structure of the Activity Environment | Structure of the Activity Environment | 2 (0.5) |
| I praise my child for being physically active or for participating in sports. | Emotional Support | Expressing Positive Emotions | 2 (0.5) |
| I support my child in being physically active. | Emotional Support | Parental Encouragement | 2 (0.5) |
| I use sport/physical activity as a form of family recreation (e.g., going on bike rides together, hiking, skating). | Structure of the Activity Environment | Structure of the Activity Environment | 2 (0.5) |
| My child has a television in his/her bedroom. | Structure of the Activity Environment | Structure of the Activity Environment | 2 (0.5) |
| I allow my child to be less active when we are on vacation. (*) | Parental Control | Child Control | 1 (0.3) |
| I allow my child to skip physical activity or sports when s/he wants to. | Parental Control | Child Control | 1 (0.3) |
| I change my schedule so that I can exercise with my child. | Autonomy Promotion | Modeling | 1 (0.3) |
| I encourage my child to be less sedentary. | Emotional Support | Parental Encouragement | 1 (0.3) |
| I encourage my child to use resources in our neighborhood to be active (such as the park, community centres, or school facilities). | Emotional Support | Parental Encouragement | 1 (0.3) |
| I go for walks with my child. | Autonomy Promotion | Modeling | 1 (0.3) |
| I have a rule that my child must do housework/chores before going out to be active. (*) | Structure of the Activity Environment | Structure of the Activity Environment | 1 (0.3) |
| I have to nag my child to be physically active or more active. (*) | Parental Control | Pressure to be Active | 1 (0.3) |
| I intentionally restrict activities that promote violence (guns, fighting). (*) | Structure of the Activity Environment | Structure of the Activity Environment | 1 (0.3) |
| I involve my child in active chores and yard work around the house. | Structure of the Activity Environment | Structure of the Activity Environment | 1 (0.3) |
| I keep track of the amount of physical activity or exercise my child gets. | Structure of the Activity Environment | Monitoring | 1 (0.3) |
| I practice active habits with my child (e.g. parking far from the door, taking the stairs). (*) | Structure of the Activity Environment | Structure of the Activity Environment | 1 (0.3) |
| I prohibit my child from playing certain sports. (*) | Parental Control | Restriction | 1 (0.3) |
| I provide my child with physical activity options from which my child can choose. | Autonomy Promotion | Autonomy Support | 1 (0.3) |
| I show an interest in my child's sport. | Emotional Support | Expressing Positive Emotions | 1 (0.3) |
| I show my child that I am angry when s/he exercises too much. | Parental Control | Expressing Negative Emotions | 1 (0.3) |
| I spend time teaching my child how to play a sport or do certain physical activities. | Informational Support | Teach/Reason | 1 (0.3) |
| I tell my child how much I like to exercise, be physically active and/or play sports. | Emotional Support | Expressing Positive Emotions | 1 (0.3) |
| I tell my child s/he is doing well in physical activities or sports. | Emotional Support | Expressing Positive Emotions | 1 (0.3) |
| I tell my child that other kids will laugh at or bully him/her if s/he participates in certain sports or activities. (*) | Parental Control | Expressing Negative Emotions | 1 (0.3) |
| I tell my child to exercise or play sports. | Parental Control | Pressure to be Active | 1 (0.3) |
| If the weather is nice, my child knows that s/he is expected to play outside. | Structure of the Activity Environment | Structure of the Activity Environment | 1 (0.3) |
| My child must be supervised when s/he (watches TV/ uses the internet). | Structure of the Activity Environment | Structure of the Activity Environment | 1 (0.3) |
| My child sees me doing active yard and house work. | Informational Support | Modeling | 1 (0.3) |
| When the weather is nice, I encourage my child to play outside. | Emotional Support | Parental Encouragement | 1 (0.3) |

* New parenting items not found in the literature
